# Potential Use of the Maillard Reaction for Pharmaceutical Applications: Gastric and Intestinal Controlled Release Alginate-Albumin Beads

**DOI:** 10.3390/pharmaceutics11020083

**Published:** 2019-02-15

**Authors:** Mouhamad Khoder, Henry K. Gbormoi, Ali Ryan, Ayman Karam, Raid G. Alany

**Affiliations:** 1 Drug Discovery, Delivery and Patient Care (DDDPC) Theme, School of Life Sciences, Pharmacy and Chemistry, Kingston University London, Kingston Upon Thames, London KT1 2EE, UK; kgbormoi@gmail.com (H.K.G.S.); A.Ryan@kingston.ac.uk (A.R.); R.Alany@kingston.ac.uk (R.G.A.); 2 Liberia Medicines & Health Products Regulatory Authority (LMHRA), Monrovia, Liberia; 3 Institut de Chimie des Milieux et Matériaux de Poitiers, Université de Poitiers, Centre National de la Recherche Scientifique, ENSIP, TSA 51106 Poitiers, France; ayman.karam@univ-poitiers.fr; 4 School of Pharmacy, The University of Auckland, Auckland 1142, New Zealand

**Keywords:** Maillard reaction, alginate, bovine serum albumin, beads, controlled release, release rate, release order, indomethacin, ciprofloxacin

## Abstract

In this study, bovine serum albumin (BSA) and alginate (ALG) conjugates were synthesized by the Maillard reaction in order to evaluate their potential to develop controlled release drug delivery systems. The progress of the Maillard reaction was evidenced using ultraviolet (UV) absorbance, determination of BSA remaining free amino groups, and sodium dodecyl sulfate polyacrylamide gel electrophoresis (SDS-PAGE). BSA-ALG conjugates possessed enhanced and tunable viscosity, foamability and foam stability. Foam generated from BSA-ALG conjugate solution was used to prepare floating gastroretentive calcium ALG beads. Unlike traditional ALG beads, BSA-ALG foam beads were able to float and sustain the ciprofloxacin (CIP) release in gastric medium. Interestingly, intestinal beads made of ALG, BSA-ALG physical mixture and BSA-ALG conjugate resulted in different release rates and orders of indomethacin (IND) in simulated intestinal fluids; while beads based on a physical mixture of BSA-ALG resulted in a first order sustained release profile, both systems based on ALG and BSA-ALG conjugate displayed zero order sustained release profiles with IND being released at a slower rate from the conjugate beads.

## 1. Introduction

The browning reaction is a natural non-enzymatic process by which the carbonyl groups of reducing sugars react with the free amino groups of proteins, resulting in a distinctive brown color [1]. Although the reaction itself occurs spontaneously, it could be significantly accelerated under specific conditions, e.g., temperature, humidity and pH. Louis Maillard was the first to describe this reaction in 1912 when he observed brown pigment being formed during a heating reaction between sugars and proteins. Since then, the Maillard reaction products have been widely applied, namely in the food industry, to conjugate sugars with proteins in order to create proteins with novel functionalities and different flavors, aromas, and textures [2,3,4,5,6]. However, the pharmaceutical applications of the Maillard reaction remain limited, although some recent studies have reported the development of nanoparticulate drug delivery systems based on the self-assembly of polysaccharide-protein Maillard conjugates [7,8,9,10]. The limited applications of the Maillard reaction in pharmaceutical area could be attributed to the uncontrolled nature of this reaction that results in products of low purity. In other words, Maillard products might not meet the higher standards required for pharmaceutical excipients. However, the natural and biocompatible characteristics of its raw materials and the fact that no toxic reagents or solvents are required in this reaction might suggest its safe applications for pharmaceutical purposes, namely for the oral route of administration.

Modifying the drug release profile is still one of the main areas of research in formulations science. Indeed, modifying oral drug release improves the therapeutic index by maintaining the plasma drug concentration within the therapeutic window or by targeting a specific area within the gastrointestinal tract. Furthermore, this helps reduce the dose and the dosing frequency, minimizing the side effects, and therefore enhancing patients’ compliance [11]. Depending on the drug absorption windows and/or its site of action, the drug release could be designed to take place in a controlled manner in the gastric, intestinal or even colonic segments. Gastroretentive drug delivery systems have been developed to sustain drug release within the stomach to target a local disease or sustain the drug release and absorption within its absorption window, i.e., the upper gastrointestinal tract [12]. On the other hand, sustaining drug release within the intestine is possible for drugs with wider absorption windows.

Polysaccharides, such as alginate (ALG) and cellulosic derivatives, have been traditionally used as controlled release agents [13]. However, in many cases, the hydrophilic characteristics of these polymers might lead to a fast swelling and subsequently a fast first order release. Conjugating polysaccharides with proteins seem appealing to modulate and/or tailor drug release from polysaccharide matrices. The higher the molecular weight, the higher viscosity of the resulting polysaccharide-protein conjugates [4,5]; those could be employed to slow down the release process. Similarly, the significant enhancement in emulsifying and foaming capacity of polysaccharide-protein conjugates [4,5,12,14] could be utilized to design floating systems for gastroretentive drug delivery. Beside the electrostatic attraction, the Maillard reaction is one of the very few non-enzymatic reactions that allow the formation of polysaccharide-protein conjugate [15].

Thanks to its good solubility in acidic medium, ciprofloxacin (CIP) is well absorbed from the stomach and duodenum. Therefore, it has been suggested as a good model drug in a gastroretentive drug delivery system [16]. Indomethacin (IND) is a non-steroidal anti-inflammatory drug that has been widely in the treatment of rheumatoid arthritis. Because of its weak acidic nature (pKa 4.5), IND is poorly soluble in the acidic medium of the stomach and rapidly dissolved in the intestinal medium [17]. IND oral administration is associated with severe side effects that limit its clinical application. Therefore, formulating IND in a sustained release system has been considered to reduce the dosing frequency, reduce the side effects, and improve the overall patient compliance [18].

The aim of the present work is to synthesize ALG and bovine serum albumin (BSA) conjugates via the Maillard reaction in order to evaluate their potential use in developing gastric and intestinal drug delivery systems with modulated release mechanisms. The release profiles of CIP and IND in simulated gastric and intestinal fluids will be established and compared with those obtained from matrices based on the native unreacted polymers. 

## 2. Materials and Methods 

### 2.1. Materials

ALG, BSA lyophilized powder, sodium bicarbonate, 2,4,6-trinitrobenzenesulfonic acid (TNBS), CaCl_2_ and 4-(2-Hydroxyethyl) piperazine-1-ethanesulfonic acid sodium salt (HEPES), CIP and IND were all purchased from Sigma-Aldrich, Dorset, UK. Sodium dodecyl sulphate was purchased from ACROS, Leicestershire, UK.

The British Pharmacopeia standard was used to prepare the simulated gastric medium (SGM) and simulated intestinal medium (SIM) as follows:

- SGM: 0.1M hydrochloric acid (pH 1.2).
- SIM: 2.4g HEPES and 8.5g NaCl were dissolved in 1 L distilled water to obtain concentrations of 9.2 mM and 150 mM respectively. The pH was then adjusted to 6.5 using diluted HCl or NaOH solutions as required.

### 2.2. Preparation of Bovine Serum Albumin-Alginate (BSA-ALG) Conjugates

Briefly, BSA (1.5 g) and ALG (3 g) were dissolved in 100 mL deionized water to yield 2 to 1 BSA:ALG molar ratio. The mixture was then frozen in liquid nitrogen before being freeze-dried (VirTis Benchtop Pro, Winchester, UK) for 72 h. The dry material was transferred into a desiccator with saturated NaCl solution to control the relative humidity (79%) and incubated at 60 °C in an oven (Binder, Germany) for 0, 3, 16 and 24 h. 

### 2.3. Characterization of BSA-ALG Conjugates

#### 2.3.1. Ultraviolet (UV) Absorbance Measurement 

The browning degree resulting from the Maillard reaction was quantified by measuring the absorbance of the BSA-ALG conjugates solution (6.66 mg/mL) at 420 nm using an ultraviolet-visible (UV-Vis). spectrophotometer (GENESYS G10S, China) [19]. All measurements were conducted in triplicate and the results were presented as mean ± standard deviation.

#### 2.3.2. Free Amino Group Determination

The percentage of the post-reaction remaining free amino groups of BSA was determined as described elsewhere using the TNBS reagent method with a few minor changes [4,20]. Briefly, 1 mL TNBS (0.1% *w*/*v*) and 1 mL NaHCO_3_ (4% *w*/*v*, pH 8.5) were added to 1 mL BSA-ALG conjugate solutions (0.5 mg/mL) and incubated at 40 °C for 2 h. Then, 1 mL sodium dodecyl sulfate (SDS) solution (10% *w*/*v*) was added to prevent the precipitation of BSA, followed by the addition of 0.5 mL HCl (1M). The absorbance was then measured at 344 nm using a spectrophotometer (GENESYS G10S, China) blanked with a control sample treated as mentioned above with 1 mL distilled water used instead of the BSA-ALG conjugate solution. 

#### 2.3.3. Sodium Dodecyl Sulfate Polyacrylamide Gel Electrophoresis (SDS-PAGE)

BSA-ALG conjugates (20 mg/mL) and BSA (10 mg/mL) solutions were prepared and boiled for 10 min. The electrophoresis separation was then conducted for 45 min using 8% acrylamide separating gel and 5% stacking comprising 0.1% SDS under a constant voltage of 185 V. Coomassie Brilliant blue solution (45% methanol and 10% acetic acid) was used to stain the gels for 30 min, before being destained with a 10% ethanol, 10% acetic acid solution for 24 h. 

#### 2.3.4. Rheological Analysis

The viscosity of BSA-ALG conjugate solutions (0.5 g/L) was measured using a rotating Brookfield viscometer (Brookfield, Inc., New York, NY, USA). The viscosity values, expressed in mPa·s, were obtained at a speed of 100 rpm using a spindle (size 5). 

#### 2.3.5. Foamability and Foam Stability Measurement

The foamability and the foam stability of BSA-ALG conjugate solutions were measured based on a method reported elsewhere [21,22]. BSA-ALG conjugates (0.5 g) were dissolved in 25 mL deionized water. The solutions were then transferred into a 50 mL measuring cylinder and homogenized at 30,000 rpm for 3 min using a vertical homogenizer (ScilogexRocky Hill, CT, USA). The volume of foam formed and the original volume of the solution was used to calculate foamability following:

$$\mathrm{Foamability}=\frac{\left(\mathrm{Foam}\;\mathrm{volume}\;\left(\mathrm{mL}\right)\right)}{\left(\mathrm{Solution}\;\mathrm{volume}\;\left(\mathrm{mL}\right)\right)}$$

The half-life (*t*_1/2_) of the foam was also recorded to assess the foam stability. The foam *t*_1/2_ is the time in minutes taken for the initial foam volume to halve in volume after its formation. 

### 2.4. Beads Preparation, Characterization and Drug Release Studies

#### 2.4.1. Floating Gastro-Retentive Beads Preparation

0.45 g BSA and ALG conjugates reacted for 0 or 24 h (BSA-ALG-0h and BSA-ALG-24 h) were separately dissolved in 10 mL distilled water to yield a solution containing 3% (*w*/*v*) ALG and 1.5% (*w*/*v*) BSA. As a control formulation, a 3% (*w*/*v*) ALG solution was also prepared in 10 mL distilled water. To each of the aforementioned solutions, 50 mg CIP (0.5% *w*/*v*) was then added slowly while stirring to avoid aggregation formation before being subjected to a 30,000 rpm homogenizing speed for 3 min in order to generate foams. The foam produced was then added dropwise using a 10 mL syringe into a 250 mL CaCl_2_ solution (10% *w*/*v*). The resulting gel-like beads were kept in the CaCl_2_ solution stirred at 400 rpm for 30 min to complete the ALG cross-linking with Ca^+2^ ions. The foamy beads were then rinsed for one minute in 250 mL distilled water using the magnetic stirrer at the same speed mentioned above. Following the rinsing process, the beads were collected using a filter paper and dried at 40 °C for 24 h using a traditional oven (Binder, Germany) [23]. 

#### 2.4.2. Intestinal Beads Preparation

0.45 g conjugates (BSA-ALG-0 h and BSA-ALG-24 h) were separately dissolved in 10 mL distilled water to obtain a 3% (*w*/*v*) ALG and 1.5% (*w*/*v*) BSA solution. As a control formulation, a 3% (*w*/*v*) ALG solution was prepared in 10 mL distilled water. To each of the BSA-ALG conjugate solution, 75 mg IND (0.75% *w*/*v*) was added and mixed slowly to avoid the formation of drug aggregates. The obtained system was then added dropwise using a 10 mL syringe into a 250 mL of 10% (*w*/*v*) CaCl_2_ solution and gently stirred using a magnetic stirrer at 400 rpm for 30 min before being collected, rinsed for 1 min in 250 mL deionized water, and dried for 24 h at 40 °C in a traditional oven (Binder, Germany).

#### 2.4.3. Scanning Electron Microscopy (SEM)

Both gastric and intestinal beads were examined under scanning electron microscopy (SEM) using Zeiss Evo50 electron microscope (Oxford instrument, Abingdon-on-Thames, UK). Prior to imaging, the beads surfaces were coated with gold and the electron microscope was operated at an accelerating voltage of 30 kV under low-vacuum mode.

#### 2.4.4. Drug Release Study

The in-vitro release of CIP and IND from the ALG, BSA-ALG-0h and BSA-ALG-24 h gastric and intestinal beads were evaluated in 500 mL SGM and SIM, respectively. A USP Dissolution Apparatus 2–Paddle (DIS-6000, Copley Scientific, Nottingham, UK) set at a temperature of 37 ± 0.5 °C and a stirring rate of 100 rpm were used to perform the release studies. The beads (15 beads of each formulation) were separately placed in the dissolution medium and aliquots of 3 mL were aspirated periodically at suitable time intervals (5, 10, 20, 40, 60, 90, 120, 150 and 180 min for gastric beads) and (5, 10, 20, 40, 60, 90, 120, 150, 180, 240 and 300 min for intestinal beads) and filtered through 0.45 μm Milipore filters before being analyzed, at 278 nm for CIP and at 320 nm for IND, using a UV spectrometer (JENWAY, Dunmow, Essex, UK). Standard calibration curves of CIP and IND were created in SGM and SIM respectively (*R*^2^ = 0.995 for CIP, *R*^2^ = 0.995 for IND). 

#### 2.4.5. Statistical Analysis

Statistical significance was determined using one-way analysis of variance (ANOVA) and Student’s *t*-tests as appropriate. All experiments were performed in triplicate and values were expressed as the mean ± standard deviation. Values of *p* < 0.05 were considered statistically significant. 

## 3. Results

### 3.1. Characterisation of ALG-BSA Conjugates

**Figure 1:**
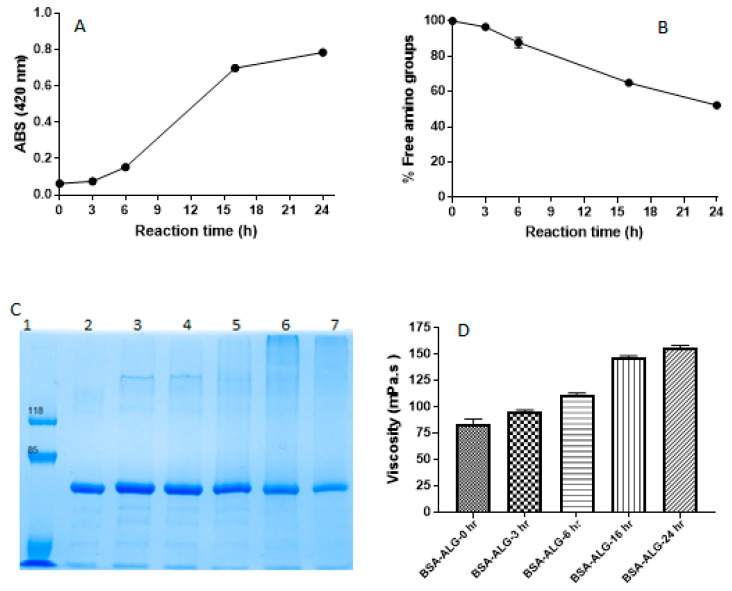
Changes in absorbance at 420 nm (**A**) and the remaining free amino groups (%) (**B**) of BSA-ALG conjugates (0, 3, 6, 16, and 24 h). (**C**) **8%** sodium dodecyl sulfate polyacrylamide gel electrophoresis (SDS–PAGE) pattern of protein marker (lane 1), BSA (lane 2), and bovine serum albumin-alginate (BSA-ALG) conjugates reacted for 0, 3, 6, 16 and 24 h (lanes 3 to 7, respectively). (**D**) The viscosity (mPa·s) of BSA-ALG conjugates (0, 3, 6, 16, and 24 h) solution. (Mean ± SD, *n* = 3, error bars within the data point symbol).

Figure 1A represents the changes in the conjugates absorbance (at 420 nm) against the reaction time; as the Maillard reaction progressed, browning color intensified significantly (*p* < 0.05). This increase in browning color intensity was also accompanied with a progressive decrease in the percentage of remaining BSA free amino groups, as Figure 1B points out. Approximately 48% of BSA free amino groups were lost after 24 h reaction time.

Figure 1C shows the sodium dodecyl sulfate polyacrylamide gel electrophoresis (SDS-PAGE) pattern of BSA and BSA-ALG conjugates. Untreated BSA revealed a single band in the range of 60 kDa, reflecting the molecular weight of this molecule [24]. It is worth mentioning that the intensity of BSA bands decreased steadily as the Maillard progressed (lanes 3–7) [4,5]. This was also associated with the appearance of a smear on lane 6 (BSA-ALG-16 h), rather than a narrowed band, indicating the formation of products of a wide range of high molecular weight. A similar observation was reported by Choi et al. where ovalbumin and dextran Maillard conjugates were obtained [25]. However, the smear of the conjugate obtained after 24 h of reaction was of reduced intensity compared to that of 16 h reaction conjugate.

The viscosity values of BSA-ALG conjugates solutions are shown in Figure 1D; as the Maillard reaction progressed, the viscosity of the Maillard products solutions increased incrementally and significantly. While the viscosity of the solution prepared based of the unreacted mixture (BSA-ALG-0h) was around 80 mPa·s, the viscosity of the BSA-ALG-16 and BSA-ALG-24 h solutions have almost doubled (147 mPa·s (*p* < 0.05) and 155 mPa·s (*p* < 0.05), respectively) (Figure 1D). These results are in good accordance with those reported previously [26].

**Figure 2:**
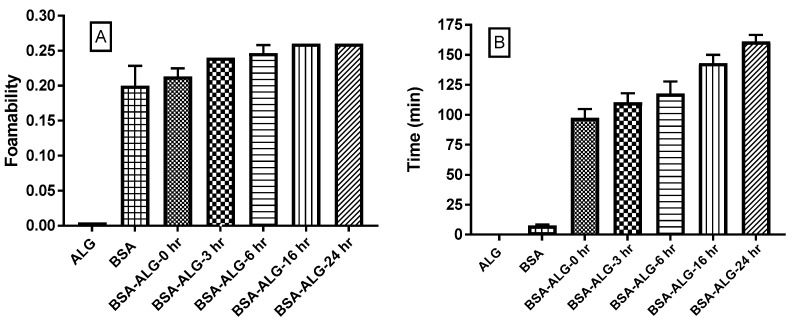
The foamability (**A**) and foam stability (min) (**B**) of ALG, BSA, and BSA-ALG BSA-ALG conjugates (0, 3, 6, 16, and 24 h) solutions.

Figure 2A represents the foamability of ALG, BSA, and BSA-ALG conjugate solutions. While BSA displayed a high foamability (up to 0.2), the ALG solution itself did not foam at all. However, all BSA-ALG conjugates formed foams with a progressive and significant (*p* < 0.05) increase in the foamability as the reaction progressed. Figure 2B represents the foam stability expressed in the foam’s half-life in minutes against the reaction times. Indeed, the BSA foam was not stable (*t*_1/2_ < 8 min), however those obtained after the addition of ALG were considerably more stable with a progressive increase (*p* < 0.05) in the foam’s half-life as the reaction progresses (up to 150 min for BSA-ALG24h solution) (Figure 2B).

### 3.2. Beads Preparation, Characterization and Drug Release Studies

**Figure 3:**
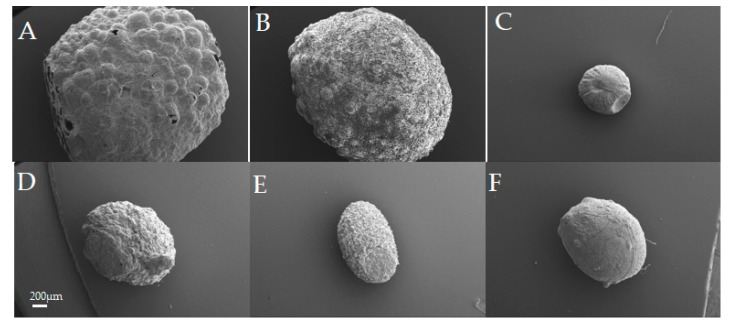
The scanning electron microscope (SEM) images of gastric and intestinal beads: (**A**–**C**) BSA-ALG-24 h, BSA-ALG-0 h, and ALG gastric beads respectively. (**D**–**F**) BSA-ALG-24 h, BSA-ALG-0 h, and ALG intestinal beads, respectively (scale bar 200 µm).

Gastroretentive beads were prepared by the ionotropic gelation of ALG solution in CaCl_2_ solution. Three formulations were considered in this stage of the current study; BSA-ALG-0 h, BSA-ALG-24 h and ALG 3% solution as a control formulation. The high foamability and foam stability of BSA-ALG systems (physical mixture (0 h) and 24 h conjugates) allowed the formation of foamy porous alginate beads with an average size of 1 to 2 mm. (Figure 3A,B and Figure S1A,B). On the other hand, beads obtained from a pure ALG solution, which failed to foam (Figure 3C and Figure S1C), were significantly smaller (*p* > 0.5mm) and did not possess the foamy nature (Figure 3C and Figure S1C). 

The floating test revealed that both BSA-ALG-0 h and BSA-ALG-24 h foam beads had the ability to float in gastric medium up to 48 h. However, those prepared using alginate alone sank immediately in the gastric medium (Table 1).

**Figure 4:**
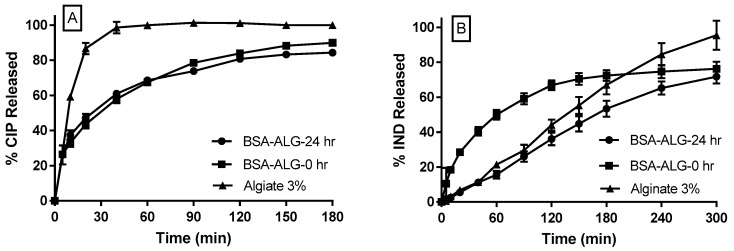
(**A**) Release profiles of ciprofloxacin (CIP) in SGM from ALG3%, BSA-ALG-0 h and BSA-ALG-24 h gastric beads. (**B**) Release profiles of indomethacin (IND) in simulated intestinal medium (SIM) from ALG3%, BSA-ALG-0 h and BSA-ALG-24 h intestinal beads.

Figure 4A shows CIP release profiles from ALG, BSA-ALG-0 h and BSA-ALG-24 h beads in SGM. Indeed, ALG beads resulted in an immediate release profile of CIP with approximately 85% released within the first 20 min. However, BSA-ALG-0 h and BSA-ALG-24 h foam beads released the drug at a rate that was significantly slower (*p* < 0.05) with approximately 35% of CIP released in the first 20 min and less than 80% released within 2 h. Nevertheless, there was no significant difference in the release profiles of CIP from both foam beads formulations (*p* > 0.05).

Intestinal beads were prepared using the same technique (i.e., ionotropic gelation) and formulations mentioned above (i.e., BSA-ALG-0 h, BSA-ALG-24 h and ALG 3% as a control) without any foaming step being involved in the process. All the formulations produced traditional beads (i.e., no foamy structure) of an average size of 1 mm, with those containing BSA (BSA-ALG-24 h, BSA-ALG-0 h) being slightly rougher than ALG 3% beads (Figure 3D–F and Figure S1D–F).

Figure 4B represents the release profile of IND in SIM form BSA-ALG-0 h, BSA-ALG-24 h and ALG beads. ALG beads release IND slowly and consistently over 5 h, following a zero order kinetics. Interestingly, formulating the ALG with BSA, reacted or not, affected both the release rates and orders of IND in SIM. Indeed, IND release from BSA-ALG-0h was significantly faster (*p* < 0.05) than from ALG beads with approximately 60% of IND released during the first 90 min. A slower release rate was then obtained resulting in first order release kinetics. In contrast, IND release from BSA-ALG-24 was overall significantly slower than that from ALG beads (*p* < 0.05) with a zero order kinetics, comparable to that obtained from ALG beads.

## 4. Discussion

In the present work, BSA-ALG conjugates were prepared using the Maillard reaction with the aim of exploring their potential as controlled release agents.

The size and percentage of free amino groups of the BSA-ALG conjugates produced were determined. As the products of the Maillard reaction display a distinctive brown colour, it was possible to assess the reaction progress quantitatively by reading the product absorbance at 420 nm. In fact, as the Maillard reaction progresses, browning intensified significantly (*p* < 0.05) indicating the Amadori compounds’ degradation and the production of the intense brown coloured melanoidins; the final product of the Maillard reaction. This progressive increase in browning colour intensity was also accompanied with a progressive decrease in the BSA free amino groups percentage (Figure 1A,B) that might also indicate the conjugation of these groups with the ALG carboxylate groups resulting in the brown colour [4,14]. The formation of BSA-ALG conjugates was confirmed via SDS-PAGE. The progressive decrease in the intensity of BSA bands is thought to be attributed to the conjugation of BSA with ALG, resulting in products of higher molecular weights [4,5]. Since the molecular weight of ALG molecules could range between 32 to 400 kDa and the Maillard reaction is an uncontrolled reaction, the BSA-ALG conjugates obtained are expected to have wide range of molecular weights. This was displayed as smeared, rather than discrete, bands on the SDS-PAGE [25] after 16 h. The reduced intensity smear produced by BSA-ALG-24 h conjugate, compared with that of the BSA-ALG-16 h, could be explained by the reduced solubility of the conjugate in water; indeed, this conjugate solution contained a noticeable amount of insoluble particles.

The progressive formation of BSA-ALG conjugates with higher molecular weights was also evidenced by the significant, progressive and controlled enhancement of the conjugate solution viscosity that is thought to be of paramount importance for tailoring different sustained release rates [27]. The swelling-diffusion drug controlled release mechanism relies mainly on the viscosity of the diffusion outer layer formed around the solid dosage form; higher viscosity yields slower release rates [28]. Likewise, the foamability and foam stability of BSA-ALG conjugate solutions were significantly and progressively enhanced via the Maillard reaction. In fact, it is well established that BSA has an intrinsic emulsifying capacity that is expected to result under vigorous agitation in the formation of an air in water emulsion; i.e., foam. However, due to the low viscosity of BSA solution (data not shown), the obtained foam collapsed rapidly. The addition of ALG to the solution enhanced the system viscosity, allowing the formation of foams with better stability. As the Maillard reaction was associated with viscosity improvement, a comparable enhancement in the foam stability was also recorded.

When ALG solution is added dropwise to certain divalent cation solutions, such as calcium and zinc, the cations diffuse inside the ALG droplets and crosslink the ALG chains causing ALG gelation and the formation of beads [13,29]. Dispersing a drug in ALG solution would result in ALG beads loaded with drug. The high foamability and foam stability of BSA-ALG systems (physical mixture (0 h) and 24 h conjugates) suggested the possible creation of ALG beads using the foam as the starting system instead of a traditional solution. Actually, the foams obtained using BSA-ALG-0 h and BSA-ALG-24 h were stable enough to allow the formation of foamy beads. However, as the pure ALG solution failed to foam, the obtained beads were rather traditional and did not possess the foamy nature. It is worth mentioning that ALG has kept its crosslinking capacity even after 24 h of reaction with BSA, suggesting that enough unreacted ALG carboxylate groups were still available even after 24 h of reaction for crosslinking with Ca^+2^. Due to its dissolution profile, CIP has a short absorption window that is located within the upper gastrointestinal tract (stomach and duodenum) [23,30]. Consequently, extending CIP release over the entire small intestine region, using traditional controlled release systems, is not conceivable. The gastro-retentive dosage forms are designed to extend the dosage form residence time in the stomach. This is why a gastroretentive beads were suggested to prolong the CIP release within its absorption window (i.e., the upper gastrointestinal tract). Having a low density, CIP-loaded foamy beads were suggested as a gastric floating system; in this system, the BSA played the role of the foaming “floating” agent whereas the ALG has been used as a matrix forming and controlled release agent. The very low density of foam beads (BSA-ALG-0 h and BSA-ALG-24 h) resulted in a floating system, considering that both ALG and BSA are not soluble in the acidic gastric medium. ALG-based systems are classified under swelling/diffusion controlled release systems [13]. Given that ALG does not swell in acidic medium (i.e., SGM), the CIP release from ALG-based beads were rather immediate. Adding the BSA to the formulations, whether they reacted or not, slowed down significantly the CIP release rate. In fact, it has been reported that BSA could undergo a severe coacervation in acidic medium [31], which is thought to be the main reason for decreasing CIP release rate in this study. Again, given that ALG-BSA conjugates could not swell in the gastric medium, the swelling/diffusion mechanism is unlikely to be involved in controlling the drug release. This might also explain the fact that no significant differences were noticed when comparing CIP release from BSA-ALG-0 h and BSA-ALG-24 h beads in spite of the enhanced viscosity discussed above. Given that the gastric residence time is 1 to 2 h [32], gastric beads displayed an excellent buoyancy ability. However, the release rate of CIP from the gastric beads was rather fast with approximately 80% released in 3 h. Intestinal beads were designed to sustain IND release in the intestinal medium. ALG 3% *w*/*v* beads sustained the release of IND in SIM for up to 5 h. This followed the typical zero order release profile of small molecules from alginate-based systems in intestinal medium [13,33]. As ALG swells considerably in intestinal medium, the controlled release of IND could be attributed the swelling/diffusion mechanism. Interestingly, adding BSA to the formulations affected significantly both the drug release rate and order. Indeed, BSA is fully soluble in water and could, to some extent, diffuse through the swollen ALG matrix. Therefore, when physically mixed with ALG (i.e., BSA-ALG-0 beads), the BSA might itself undergo a dissolution and release process leaving a channelled ALG matrix. Consequently, the IND diffusion process could be enhanced resulting in the faster first order release kinetics shown in Figure 4B. On the other hand, covalently linking BSA molecules with the ALG ones via the Maillard reaction (i.e., BSA-ALG-24 beads), prevented BSA diffusion through the ALG matrix, hindering the accelerated release of IND. Furthermore, the higher molecular weights and viscosity of the resulting conjugates are expected to generate a less porous, more viscous, and highly tortuous swollen ALG-BSA diffusion layer that could decelerate further the IND release, resulting in an overall slower zero order release profile.

## 5. Conclusions

In this study, the potential use of BSA-ALG Maillard conjugates as a control release agent was investigated. BSA-ALG Maillard conjugates were prepared and characterised using UV absorbance, quantification of the remaining free amino groups of BSA, viscosity measurement, and SDS PAGE. CIP-loaded gastroretentive beads were prepared by the crosslinking of BSA-ALG-0 h and BSA-ALG-24 h conjugates foams in CaCl_2_ solution. Gastric beads displayed an excellent buoyancy capacity in gastric medium and were able to sustain the CIP release time over 3 h. Interestingly, IND-loaded calcium ALG beads, made of BSA-ALG conjugates solutions, were able to modify both the drug release rate and order in intestinal fluid; while BSA-ALG-0 h beads resulted in a first order sustained release profile, those made of BSA-ALG-24 h conjugate solution displayed slower zero order sustained release profiles. Overall, the results demonstrated the potential of using protein-polysaccharide Maillard conjugate for pharmaceutical applications, namely for control release systems.

## Figures and Tables

**Table 1:** Floating time of gastroretentive beads in the simulated gastric medium (SGM).

Formulation	Floating Time in SGM
ALG 3% beads	<1 min
BSA-ALG-0 h beads	up to 48 h
BSA-ALG-24 h beads	up to 48 h

## Supplementary Materials

The following are available online at http://www.mdpi.com/1999-4923/11/2/83/s1, Figure S1. Light microscopic images of gastric and intestinal beads: (A–C) BSA-ALG-24 h, BSA-ALG-0 h, and ALG gastric beads respectively. (D–F) BSA-ALG-24 h, BSA-ALG-0 h, and ALG intestinal beads, respectively (scale bar 1 mm). Figure S2. The loading efficiency (%) of IND (A) and CIP (B) in intestinal and gastric beads respectively.
