# Supplementary material for: Potential Use of the Maillard Reaction for Pharmaceutical Applications: Gastric and Intestinal Controlled Release Alginate-Albumin Beads
Source: Pharmaceutics. 2019 Feb 15;11(2):83. doi: 10.3390/pharmaceutics11020083 (PMC6410193; doi:10.3390/pharmaceutics11020083)
Supplement: Supplementary file 1 [file pharmaceutics-11-00083-s001.pdf]

pharmaceutics

Supplementary Materials: Potential Use of the Maillard Reaction for Pharmaceutical Applications: Gastric and Intestinal Controlled Release Alginate-Albumin Beads

Mouhamad Khoder, Henry K. Gbormoi, Sr., Ali Ryan, Ayman Karam and Raid G. Alany

Light Microscopic Images

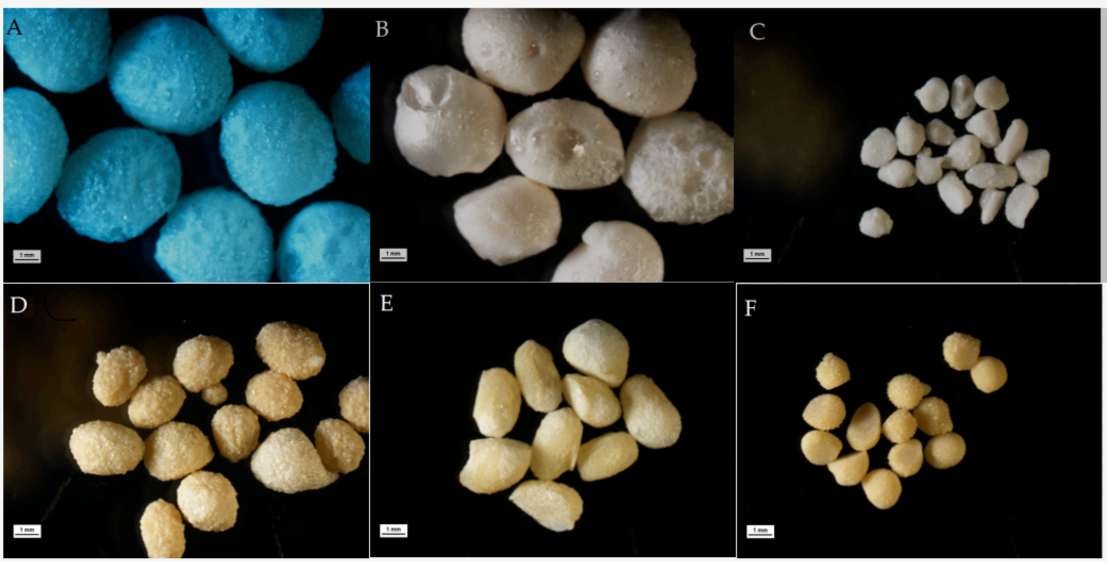

Figure S1. Light microscopic images of gastric and intestinal beads: (**A**–**C**) BSA-ALG-24 h, BSA-ALG-0 h, and ALG gastric beads respectively. (**D**–**F**) BSA-ALG-24 h, BSA-ALG-0 h, and ALG intestinal beads, respectively (scale bar 1 mm).

Loading Efficiency Studies

Five beads of each formulation were separately placed in 100 mL of dissolution medium and stirred for 24 h, followed by 30 sonication in order to ensure the complete release and dissolution of the loaded drugs. Samples were then taken, filtered and the amount of released IND or CIP was analyzed by UV spectroscopy. The loading efficiency (Figure S2) was determined according to the formula:

$$LE = \frac{Mm}{Mi} * 100$$

Where *Mm* the amount of drug released from five dried beads and *Mi* is the initial amount of drug dispersed in the ALG and BSA-ALG solutions required to form five beads.

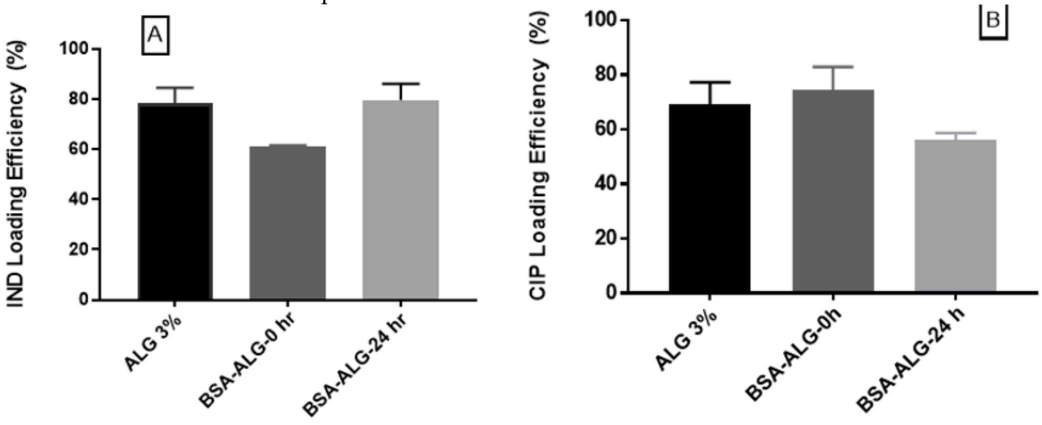

Figure S2. The loading efficiency (%) of IND (**A**) and CIP (**B**) in intestinal and gastric beads respectively.
